# Naringin Mediates Adult Hippocampal Neurogenesis for Antidepression via Activating CREB Signaling

**DOI:** 10.3389/fcell.2022.731831

**Published:** 2022-04-07

**Authors:** Chong Gao, Meiling Wu, Qiaohui Du, Jiagang Deng, Jiangang Shen

**Affiliations:** ^1^ School of Chinese Medicine, Li Ka Shing Faculty of Medicine, University of Hong Kong, Hon Kong SAR, China; ^2^ The Institute of Brain and Cognitive Sciences, School of Medicine, Zhejiang University City College, Hangzhou, China; ^3^ Guangxi Key Laboratory of Efficacy Study on Chinese Materia Medica, Guangxi University of Chinese Medicine, Nanning, China

**Keywords:** depression, adult hippocampal neurogenesis, naringin, BDNF pathway, CREB

## Abstract

The brain-derived neurotrophic factor/tropomyosin receptor kinase B/cAMP response element-binding protein (BDNF/TrkB/CREB) signaling pathway is a critical therapeutic target for inducing adult hippocampal neurogenesis and antidepressant therapy. In this study, we tested the hypothesis that naringin, a natural medicinal compound, could promote adult hippocampal neurogenesis and improve depression-like behaviors via regulating the BDNF/TrkB/CREB signaling pathway. We first investigated the effects of naringin on promoting adult hippocampal neurogenesis in both normal and chronic corticosterone (CORT)-induced depressive mice. Under physiological condition, naringin treatment enhanced the proliferation of neural stem/progenitor cells (NSPCs) and accelerated neuronal differentiation. In CORT-induced depression mouse model, naringin treatment promoted neuronal differentiation and maturation of NSPCs for hippocampal neurogenesis. Forced swim test, tail suspension test, and open field test confirmed the antidepressant and anxiolytic effects of naringin. Co-treatment of temozolomide (TMZ), a neurogenic inhibitor, abolished these antidepressant and anxiolytic effects. Meanwhile, naringin treatment increased phosphorylation of cAMP response element binding protein (CREB) but had no effect on the expression of brain-derived neurotrophic factor and phosphorylation of TrkB in the hippocampus of CORT-induced depressive mice. Co-treatment of CREB inhibitor 666-15, rather than TrkB inhibitor Cyc-B, abolished the neurogenesis-promoting and antidepressant effects of naringin. Taken together, naringin has antidepressant and anxiolytic effects, and the underlying mechanisms could be attributed to enhance hippocampal neurogenesis via activating CREB signaling.

## Introduction

Depression is a global disease burden (Rehm and Shield 2019). Different interventions, including tricyclic antidepressants, selective serotonin reuptake inhibitors (SSRIs), monoamine oxidase inhibitor, electroconvulsive therapy, exercise, lithium, and pindolol, are developed for antidepressant treatment (Cipriani et al., 2011). However, clinical effects and long-term outcomes are still unsatisfactory. Some antidepressants even induce or worsen suicidal ideation, behavior, and agitation (Morilak and Frazer 2004; Cipriani et al., 2011; Di Giovanni et al., 2016). With the rapid increase of depressive patients worldwide, seeking novel molecular targets and therapeutic strategies is important for antidepressant therapy.

Adult hippocampal neurogenesis offers an optimized neural plasticity to enhance behavioral response for antidepression (Castren and Rantamaki 2010; Duman and Li 2012). *Hippocampus* is a major brain region for learning, memory, and emotional regulation (Girardeau et al., 2017). In hippocampal dentate gyrus (DG) loci, neural stem/progenitor cells (NSPCs) undergo the self-renewal expanding, lineage commitment and mature into projective neurons, integrating into neural circuits. The impaired hippocampal neurogenesis is a major hallmark contributing to depressive and anxiety behaviors (Brezun and Daszuta 1999; Sahay and Hen 2007). In rodents, genetic enhancement of neurogenesis is sufficient to relieve depressive/anxiety mood (Culig et al., 2017). Adult hippocampal neurogenesis is orchestrated by multiple signaling molecules. Growth factors and neurotrophic factors play critical roles in the pathogenesis of depression and antidepression responses (Duman and Li 2012; Gao et al., 2017). Chronic stress decreases the brain-derived neurotrophic factor (BDNF) and inactivates its receptors, including tropomyosin receptor kinase B (TrkB) and downstream signaling-like cAMP response element-binding protein (CREB) (Luo et al., 2017; Peng et al., 2018). Antidepressant treatment elevates BDNF expression and promotes proliferation and differentiation of NSPCs and the maturation of new neurons for hippocampal neurogenesis (Masi and Brovedani 2011; Ma et al., 2017). Thus, targeting the BDNF/TrkB/CREB signaling pathway for adult hippocampal neurogenesis could be an important strategy for antidepressant drug discovery.

Traditional Chinese medicine (TCM) has a long history of treating depression-like disorders in China. Modern pharmacological studies bring opportunities to explore the active compounds from medicinal herbs for antidepressant drug discovery (Lee and Bae 2017). Tangerine peel is commonly used in the TCM formula for antidepression treatment. Naringin (4′,5,7-trihydroxy-flavonone-7-rhamnoglucoside, Ng) is a bioflavonoid identified from tangerine peel. Naringin is also ubiquitously distributed in grapefruit and related citrus species. Naringin has strong antioxidant properties based on the structural components of carbonyl group at C-4 of the C ring, hydroxyl groups at C-5 of the A ring, and at C-4′ of the B ring through rapid donation of hydrogen atoms to free radicals. Naringin possesses diverse biological activities including antioxidant (Sharma et al., 2015), antifibrosis (Chen et al., 2013), anti-inflammation (Luo et al., 2012; Nie et al., 2012), metabolic modulations (Rajadurai et al., 2006), and cholinergic transmission activation (Ben-Azu et al., 2019). Naringin was reported to protect against different brain damages in different animal models such as subarachnoid hemorrhage (Han et al., 2016), traumatic brain injury (Cui et al., 2014), cerebral ischemia-reperfusion injury (Gaur et al., 2009; Feng et al., 2018), and spinal cord injury (Rong et al., 2012). Naringin also revealed its anti-inflammation and antioxidant bioactivities in adult streptozotocin-induced hyperglycemic mice (Okuyama et al., 2018). Naringin dihydrochalcone promoted neurogenesis in APP/PS1 Alzheimer disease mice (Yang et al., 2018). Naringin has the potential for antidepressant therapy (Aggarwal et al., 2010; Ben-Azu et al., 2018; Ben-Azu et al., 2019; Oladapo et al., 2021). Yet, the underlying mechanisms for adult hippocampal neurogenesis and antidepression remain unknown. In the present study, we tested the hypothesis that naringin could promote adult hippocampal neurogenesis and improve depressant behaviors via regulating BDNF/TrkB/CREB signaling pathways.

## Methods

### Animals

C57BL/6N male adult mice (7–8 weeks) were obtained from the Laboratory Animal Unit (LAU), The University of Hong Kong. Mice were maintained in the standard environment (12 h light/dark cycle, with lights on at 8:00 a.m., ad libitum access to dry food pellets and water). All animal protocols were approved by LAU, the University of Hong Kong (CULATR No. 5090-19).

### Drug Treatments

Naringin (Ng, purity >98.3%) was extracted from *Citrus grandis* “Tomentosa” and provided by Professor Wei-Wei Su, Sun Yat-sen University, Guangzhou, China. We first investigated the effects of Ng on improving neurogenesis in both physiological and chronic corticosterone (CORT)-challenged conditions. Under physiological condition, the Ng-treated mice were orally administrated with Ng at the dosage of 50 mg/kg/d (dissolved in saline) for 5 consecutive days, while the vehicle-treated mice (Veh) received saline treatment as control. In the antidepressant experiments, we adopted a CORT-treated animal model characterized with impaired neurogenesis and depression-like behaviors and performances (Siopi et al., 2016; Gao et al., 2018b). CORT was dissolved in the solution of 0.45% beta-cyclodextrin (beta-CD, Sigma) and supplied ad libitum in drinking water (70 μg/ml, equivalent to 5 mg/kg/d) for 40 consecutive days, aiming to minimize the additional stress response and avoid the potential influence on BrdU incorporation. The reliability of the methodology was proved with the consistent inductions of the impaired neurogenesis and depressive behaviors in our previous studies (Gao et al., 2018a; Gao et al., 2018b). To study Ng’s antidepression effects, the CORT mice were treated with Ng (10, 50 mg/kg/d) by oral gavage starting from 15th day to 40th day, whereas beta-CD vehicle- and CORT-treated mice received saline treatment. The selected dosage was based on the previous protocol reported for post-stroke depression treatment (Aggarwal et al., 2010).

We then used temozolomide (TMZ, Sigma), a neurogenesis inhibitor, to explore whether the antidepressant effect was associated with neurogenesis. TMZ (25 mg/kg/d, dissolved in saline) was injected by i.p. on the first 3 days of each week continuously for 4 weeks before the forced swim test (FST) (Ma et al., 2017). The CORT-treated mice were orally treated with Ng (50 mg/kg/d) from 15th day to 40th day (Figure 3A). We also used CERB inhibitor 666-15 to explore whether CERB is a therapeutic target of Ng. In the 666-15-treated group, in addition to Ng (50 mg/kg/d) and CORT treatments, the mice were intraperitoneally injected with 666-15 (10 mg/kg/d, dissolved in saline) for five times in the interval of every 2 days (Figure 4D).

### Bromodeoxyuridine Incorporation

**FIGURE 1:**
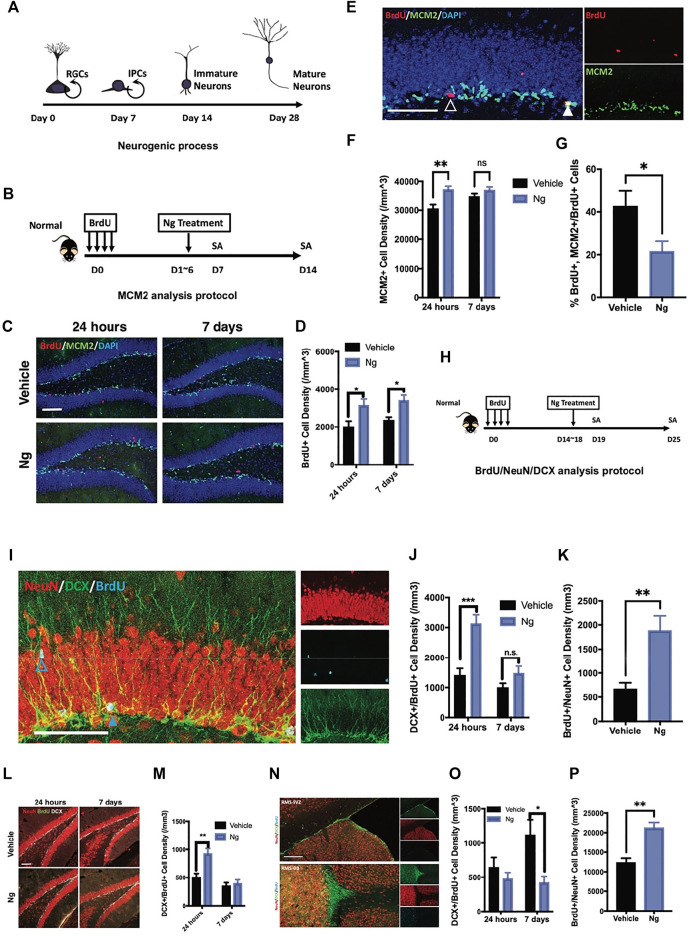
Naringin accelerates neural differentiation of NSPCs in adult mouse hippocampus. **(A)** Estimated timeline for the process of adult hippocampal neurogenesis. **(B)** Diagram for testing proliferation-promoting effects of naringin (Ng) on early phase of hippocampal NSPCs for MCM2 analytic protocol. **(C)** Representative fluorescent imaging results of dual immunostaining cells of BrdU^+^ (red) and MCM2^+^ (green) at the hippocampal DG region in vehicle- and Ng-treated mice. Scale bar, 100 μm **(D)** Statistical analysis of BrdU^+^ cells in the hippocampus of vehicle- and Ng-treated mice at 24 h and 7-days post treatment (dpt). **(E)** Representative fluorescent imaging results of MCM2^+^ (green) and BrdU^+^ cells (red) in the hippocampal DG region. DAPI was used as a nuclear label (blue). Scale bar 100 μm **(F)** Statistical analysis of MCM2^+^ cells at 24 h and 7 dpt (*n* = 6 per group, two-way ANOVA, vs. vehicle, **p < 0.01). **(G)** Statistical analysis of MCM2^+^/BrdU^+^ cells at 24 h post treatment. **(H)** Diagram for studying effects of Ng on neural differentiation of NSPCs at late phase. BrdU was injected and then Ng treatment was started at day 15 for 5 consecutive days. **(I)** Representative fluorescent imaging results of co-immunostaining cells of NeuN^+^/BrdU^+^/DCX+ in the hippocampal DG region. Scale bar, 100 μm **(J,K)** Statistical analysis of dual-positive BrdU/DCX and BrdU/NeuN cells in the hippocampal DG region, respectively. **(L)** Representative fluorescent imaging results of co-immunostaining cells of NeuN^+^/BrdU^+^/DCX^+^ in ventral hippocampus. Scale bar, 100 μm. **(M)** Statistical analysis of dual-positive BrdU/DCX and BrdU/NeuN cells in the ventral hippocampal region. **(N)** Representative fluorescent imaging results of co-immunostaining cells of NeuN^+^/BrdU^+^/DCX^+^ in olfactory bulbs (OB) and subventricular zone (SVZ) from the sagittal angle. **(O,P)** Statistical analysis of dual-positive BrdU/DCX and BrdU/NeuN cells SVZ-OB region, respectively. *n*=6 per group, *p<0.05, **p<0.01, ***p<0.001.

We adopted two protocols for detecting cell proliferation. For normal mice to explore the proliferation of NPCs, BrdU (Sigma) (100 mg/kg per time, dissolved into saline) was injected by i.p. to the mice on the first day for four times with 2.5 h intervals (Figures 1A,B). For CORT-treated mice to detect the neuronal differentiation and maturation, BrdU (50 mg/kg per day) was injected by i.p. from first day to fifth day.

### Behavioral Tests

FST, tail suspension test (TST), and open field test (OFT) were used to assess depressive and anxiety behaviors according to the protocols in our previous publication (Gao et al., 2018a). These behavior tests were video-recorded. In brief, FST) was conducted in a cylinder water tank (30 cm height × 20 cm diameters) with the water temperature at 23–25°C for 6 min. The mobility time was 4 min including struggling and free swim time. The first 2 min was defined as habituation. After FST, TST was processed. Mice were suspended on the tap-top of their tails for 6 min. Distance from the nose to the floor was 25 cm. The mobility time was recorded for learned helplessness study. OFT was performed in a white box, sized 43 × 43 cm, which lasts for 10 min. Open field was cleaned with 75% ethanol before the test. Mice were free to move in the arena with 16 separated equal squares. There were four squares in the center of the arena with about 20 cm square region. The amount of time the animals traveled in the central arena was recorded to analyze the anxiety level.

### Immunofluorescence

Mice were euthanized by cardiac perfusion with 4% paraformaldehyde (PFA). Brains were collected and post fixed in 4% of PFA for another 24 h, and then dehydrated in 30% sucrose PBS solution for 2 days. Coronal brain sections (40 μm) were prepared with freezing microtome (Leica Inc., Germany). Immunofluorescence (IF) was conducted as in our previous report (Gao et al., 2018a). After blocked with 5% goat serum (in PBS with 0.3% Triton X-100) for 1 h at room temperature, the sections were incubated with primary antibodies, as per details in the Table 1, at 4°C overnight. Then, the sections were rinsed with PBST (PBS with 1% Tween-20) for three times (5 min per time) and incubated with secondary fluorescent antibodies (details in the Table 1). After incubated with DAPI for 10 min to label nuclei, the sections were covered with mounting medium and visualized using a confocal microscope (Carl Zeiss). Image was processed by Z-stack of 20 μm with maximum projection. Positive cells were counted by projection-acquired Z-stack images. Cells were manually counted by using the plug-in “Manual counting” (http://icy.bioimageanalysis.org/plugin/manual counting).

**TABLE 1:** Resources Table

Reagent or Resource	Source	Identifier	Concentration
**Antibodies**			
anti-MCM2	Cell Signaling Technology	3,619	1:200
anti-DCX	Cell Signaling Technology	4,604	1:400
anti-NeuN	Abcam	ab104224	1:200
anti-BrdU	Abcam	ab6326	1:200
BDNF antibody	Abcam	ab108319	1:1,000
CREB antibody	Cell Signaling Technology	4,820	1:1,000
Phospho-CREB (Ser133) antibody	Cell Signaling Technology	9,198	1:1,000
Phospho-TrkB	Cell Signaling Technology	4,621	1:500
TrkB	Cell Signaling Technology	4,603	1:1,000
β-Actin antibody	Cell Signaling Technology	3,700	1:3,000
Goat anti-rabbit HRP	Cell Signaling Technology	7,074	1:5,000
Goat anti-Rat Alexa 647	Thermo Fisher	A-21247	1:800
Goat anti-Rabbit Alexa 488	Thermo Fisher	A27034	1:800
Goat anti-Mouse Alexa 568	Thermo Fisher	A-11004	1:800
**Chemicals**			
BrdU (5-bromo-2′-deoxyuridine)	Abcam	ab142567	50/100 mg/kg; 10 mg/ml in saline
Corticosterone	Abcam	ab143597	70 μg/ml; 0.45% beta-cyclodextrin in tap water
Beta-cyclodextrin	Sigma-Aldrich	C4767	0.45%
Temozolomide (TMZ)	Sigma-Aldrich	T2577	25 mg/kg; 2.5 mg/ml in saline
Cyclotraxin B	R&D System	5,062	2.5 mg/kg, 0.5 mg/ml in saline
666-15	Sigma-Aldrich	53834	10 mg/kg, 2 mg/ml in saline
Naringin	Sigma-Aldrich	71162	10/50 mg/kg; 2/10 mg/ml in saline
DAPI	Cell Signaling Technology	4,083	N/A
**Hardware & Software**			
Confocal microscope	Carl Zeiss	LSM880; LSM780	N/A
Cryostat refrigerated microtome	Leica	DOM 2008	N/A
ImageJ	NIH	https://ImageJ.nih.gov/ij/	N/A
Prism 7	GraphPad Software	https://www.graphpad.com/	N/A
**Others**			
Mounting medium	DAKO	S302380	N/A

### Western Blot

Protein concentrations were determined by using the Bio-Rad protein assay kit (Bio-Rad, United States). Equal amount of protein was applied to SDS-polyacrylamide gel electrophoresis and then transferred to polyvinylidene difluoride (PVDF) membranes (Millipore). After blocking, membranes were probed with appropriate primary antibodies (see Table 1; BDNF 1:1,000, rabbit; CREB 1:1,000, rabbit; p-CREB (Ser133) 1:1,000, rabbit; p-TrkB 1:500, rabbit; TrkB 1:1,000; β-actin 1:3,000, rabbit) for overnight incubation at 4°C. The blots were subjected to HRP-conjugated corresponding secondary antibodies (goat-rabbit-HRP, 1:5,000) at room temperature for 2 h. Protein bands were visualized by adding ECL Advance (GE Healthcare Biosciences) according to the manufacturer’s instructions. Results were analyzed by the program of Quantity One (Bio-Rad).

### Statistics

Data were presented with Mean ± SEM and analyzed with Prism 7 (GraphPad Software). Two-tailed student unpaired *t* test was applied for the comparison of two group designed studies. ANOVA was used for the comparison of multiple group designed experiments. Sidak’s multiple comparisons were performed for two group analysis in the two-way ANOVA. Turkey’s multiple comparison test was applied to analyze the differences over two groups. For all experiments, *p* < 0.05 was considered as statistically significant difference.

## Results

### Naringin Accelerates Neuronal Differentiation for Adult Hippocampal Neurogenesis in Normal Mice

We first examined the effects of naringin on adult hippocampal neurogenesis in normal mice. Figure 1A shows the experimental protocol for adult hippocampal neurogenesis covering 4 weeks from the NSPC proliferation to mature neuron formation. At early stage of neurogenesis, NSPCs turned into immature neurons after stepping into the G_0_ phase. We used exogenous cell tracer 5′-bromo-2′-deoxyuridine (BrdU) to identify cell proliferation for adult hippocampal neurogenesis. Figure 1B shows the protocol in which naringin was injected for 5 consecutive days after BrdU was injected for labeling cell proliferation. BrdU was co-immunostained with minichromosome maintenance complex component 2 (MCM2), an endogenous marker for cell proliferation. We analyzed the percentage of BrdU/MCM2 dual-positive cells for identifying cell proliferation in the hippocampus of the mice with or without naringin treatment at 24 h and 7 days. After naringin treatment, the number of BrdU^+^ cells were increased at 24 h and the increase was sustained for 7 days post treatment (dpt) (Figures 1C,D). Notably, with naringin treatment (50 mg/kg), the MCM2^+^ cells were transiently increased at 24 h, and the difference between vehicle and naringin treatment was disappeared on 7 dpt (Figures 1E,F). The naringin-treated group had lesser MCM2^+^/BrdU^+^ rates than the vehicle control group (Figures 1E,G). Thus, we suspected that naringin might accelerate the process of the newborn cells escaping from the cell cycle and committing into the differentiation stage. To verify this idea, we performed dual immunofluorescence experiments in which BrdU was co-immunostained with immature neuronal marker doublecortin (DCX) and mature neuronal marker NeuN to examine neuronal differentiation (Figure 1H). DCX is commonly expressed at the stages of proliferation for postmitotic maturation (Chen et al., 2020; Encinas and Enikolopov 2008; He et al., 2020). DCX is a surrogate marker for adult neurogenesis with a complete overlap in the localization of the polysialylated neuronal cell adhesion molecule (Kempermann et al., 2015). NSPCs could develop into the DCX^+^ progenitors and then become mature neurons with the NeuN^+^ and DCX^−^ staining in the adult hippocampus. In our study, after 24 h of naringin treatment, the mice had significantly upregulated the BrdU^+^/DCX^+^ cell density in the hippocampus (Figures 1I,J). However, it disappeared at 7 dpt of the treatment (Figures 1I,J). Instead, the mature newborn neurons with BrdU^+^/NeuN^+^ staining were remarkably increased in the hippocampus (Figure 1K). Such phenomenon was also found at the DG region (Figures 1L,M). Naringin treatment decreased the immature BrdU^+^/DCX^+^ cell density at SVZ but increased the mature neurons with BrdU^+^/NeuN^+^ staining at the OB site at 7 dpt (Figures 1O,P). It is well known that the region from the subventricular zone to olfactory bulb (SVZ-OB) is an important site of adult neurogenesis. Newborn NSPCs commit into immature neurons in SVZ and migrate to OB along rostral migratory stream (RMS) (Lledo and Valley 2016). Therefore, our results suggest that naringin could promote neuronal differentiation and induce migration of the NSPCs into SVZ-OB system, leading to adult hippocampal neurogenesis in normal mice.

### Naringin Promotes Adult Hippocampal Neurogenesis and Exerts Antidepressant Effects in Chronic Corticosterone-Treated Mice

**FIGURE 2:**
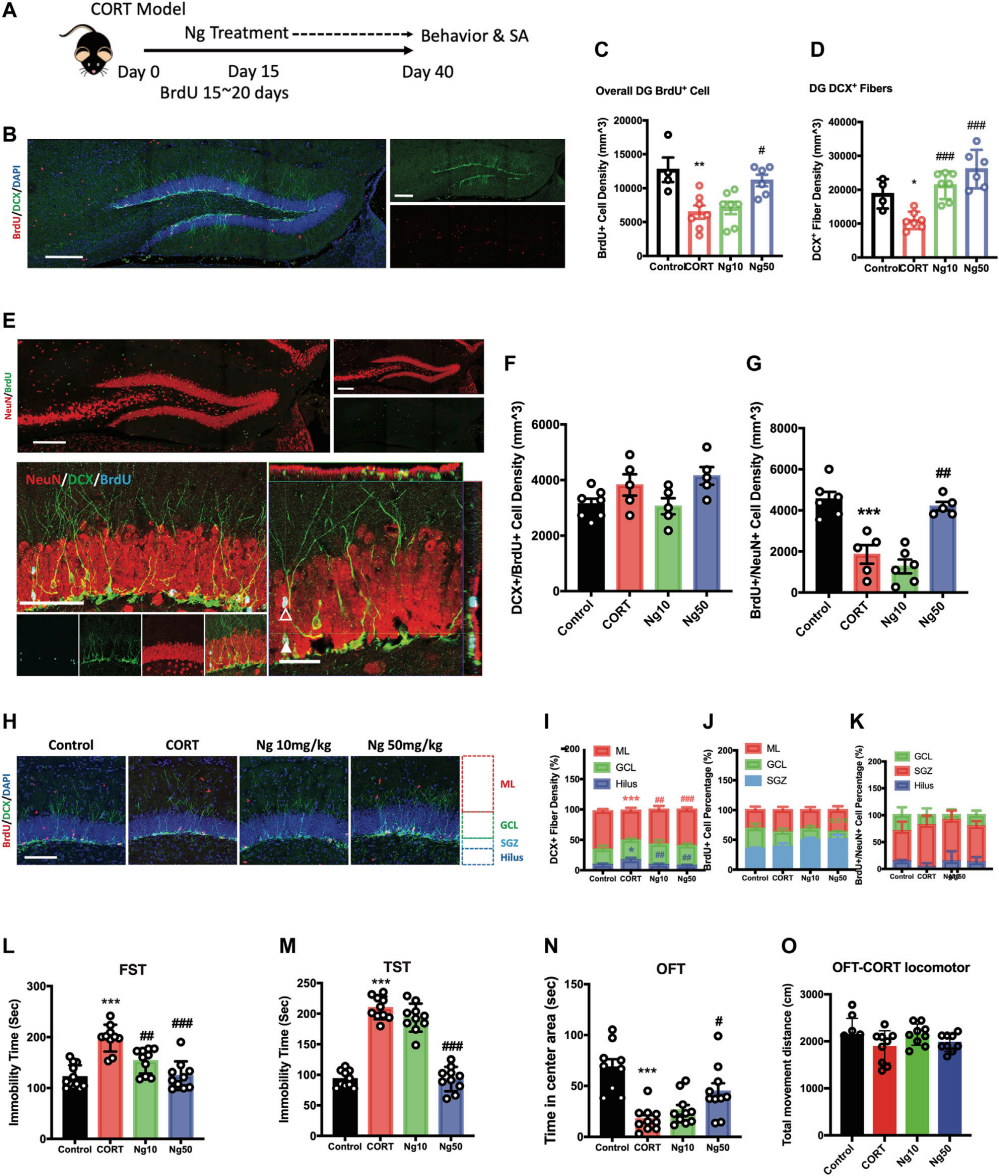
Naringin exerts antidepressant effects to improve adult hippocampal neurogenesis in corticosterone (CORT)-induced depression model. **(A)** Diagram for experimental protocol. Corticosterone (CORT) was orally administered into the mice for 40 days. **(B)** Representative fluorescent imaging showing the newly generated immature neurons with BrdU^+^ (red)/DCX^+^ (green) staining. DAPI (blue) was used for nuclear labeling. Scale bar 200 μm. **(C)** Statistical analysis of BrdU^+^ cells in the DG region (*n* = 4–7 per group, one-way ANOVA, **p < 0.01 CORT vs. Control, #*p* < 0.05 CORT vs. Ng 50 mg/kg/d). **(D)** Statistical analysis of DCX^+^ fibers in the DG region. **(E)** Representative fluorescent imaging showing newly produced immature neurons (BrdU^+^/DCX^+^) and mature neurons (BrdU^+^/NeuN^+^) in the hippocampal DG region. Upper image, new produced mature neurons in the whole DG region (red: NeuN; green: BrdU). Scale bar, 200 μm. Lower image, newly produced immature and mature neurons in the DG region (red: NeuN; green: cyan; blue: BrdU). White triangle points for newborn pre-mature neurons labeled with BrdU^+^/NeuN^+^/DCX^+^. White empty triangle points for newborn mature neurons labeled with BrdU^+^/NeuN^+^/DCX^−^. Scale bar 100 and 20 μm **(F)** Statistical analysis of DCX^+^/BrdU^+^ cells. **(G)** Statistical analysis of NeuN^+^/BrdU^+^ cells. (**H)** Representative fluorescent imaging showing the newborn immature neurons (BrdU^+^/DCX^+^), DAPI was used for nuclear labeling. Scale bar 100 μm. **(I,J,K)** Statistical analysis of distribution of DCX^+^ fibers, BrdU^+^ cells and BrdU^+^/NeuN^+^ newborn neurons in the DG region. **(L)** Statistical analysis of the immobility time in FST. **(M)** Statistical analysis of the immobility time in tail suspension test (TST). **(N)** Statistical analysis of the time spent in the central region in OFT. **(O)** Locomotor distance of the animal during the OFT. *N* = 4–10 per group, Control vs. CORT: *p < 0.05, **p < 0.01, ***p < 0.001; CORT vs. Ng #*p* < 0.05, ##*p* < 0.01, ###*p* < 0.001.

We then examined whether naringin promotes adult hippocampal neurogenesis in the CORT-induced mice charactered with dampened neurogenesis and depressive behaviors. As shown in Figure 2A, the mice were orally administrated CORT ad libitum in drinking water for 40 days. Naringin (10, 50 mg/kg/d) or vehicle treatment was orally administered to the CORT-treated mice for the period of day 15 to day 40. BrdU incorporation was performed for 5 consecutive days starting at day 15. The results showed that high dose of naringin (50 mg/kg/d) increased BrdU^+^ cells at the DG region in the CORT-treated mice (Figures 2B,C). Naringin treatment (10, 50 mg/kg/d) had no effect on the population of DCX^+^/BrdU^+^ cells (Figures 2E,F). However, the naringin treatment (50 mg/kg/d) significantly increased BrdU^+^/NeuN^+^ cells in the hippocampus of the CORT-treated mice (Figures 2E,G). These results suggest that naringin could promote the development and maturation of the BrdU^+^ newborn cells. Moreover, at the dosages of 10 and 50 mg/kg/d, naringin remarkably increased the density of DCX^+^ fibers (Figures 2B,D) and enhanced the DCX^+^ dendritic fiber expanding to molecular layer (ML) (Figures 2H,I). We then investigated the distribution of BrdU^+^ cells and BrdU^+^/NeuN^+^ cells at the molecular layer (ML), granular cell layer (GCL), and subgranular zone (SGZ). The naringin treatment had no significant effect on the migration of NSPCs in the DG region (Figures 2H,J,K). Taken together, naringin could accelerate neuronal maturation and promote the dendritic enrichment in the CORT-treated depression-like mouse model.

We next examined the effects of naringin against the CORT-induced depressive behaviors and anxiety. At the dosage of 50 mg/kg/d, naringin treatment rendered a dramatic decrease in immobility in FST and TST (Figures 2L,M), indicating the antidepressant effects. Meanwhile, naringin treatment (50 mg/kg/d) prolonged the duration of the movement in the central region in OFT, indicating the anxiolytic effects (Figure 2N). However, at the dosage of 10 mg/kg/d, naringin had no effect on the CORT-induced depressive behaviors in TST and OFT except for FST (Figure 2N). Notably, both CORT and naringin treatment had no influence on the locomotor activity (Figures 2O). Therefore, these studies suggest that naringin has antidepressant effects, and the underlying mechanisms could be associated with promoting adult hippocampal neurogenesis.

### Temozolomide Abolishes Naringin Effects on Promoting Neurogenesis and Antidepression in Corticosterone-Induced Depressive Mice

**FIGURE 3:**
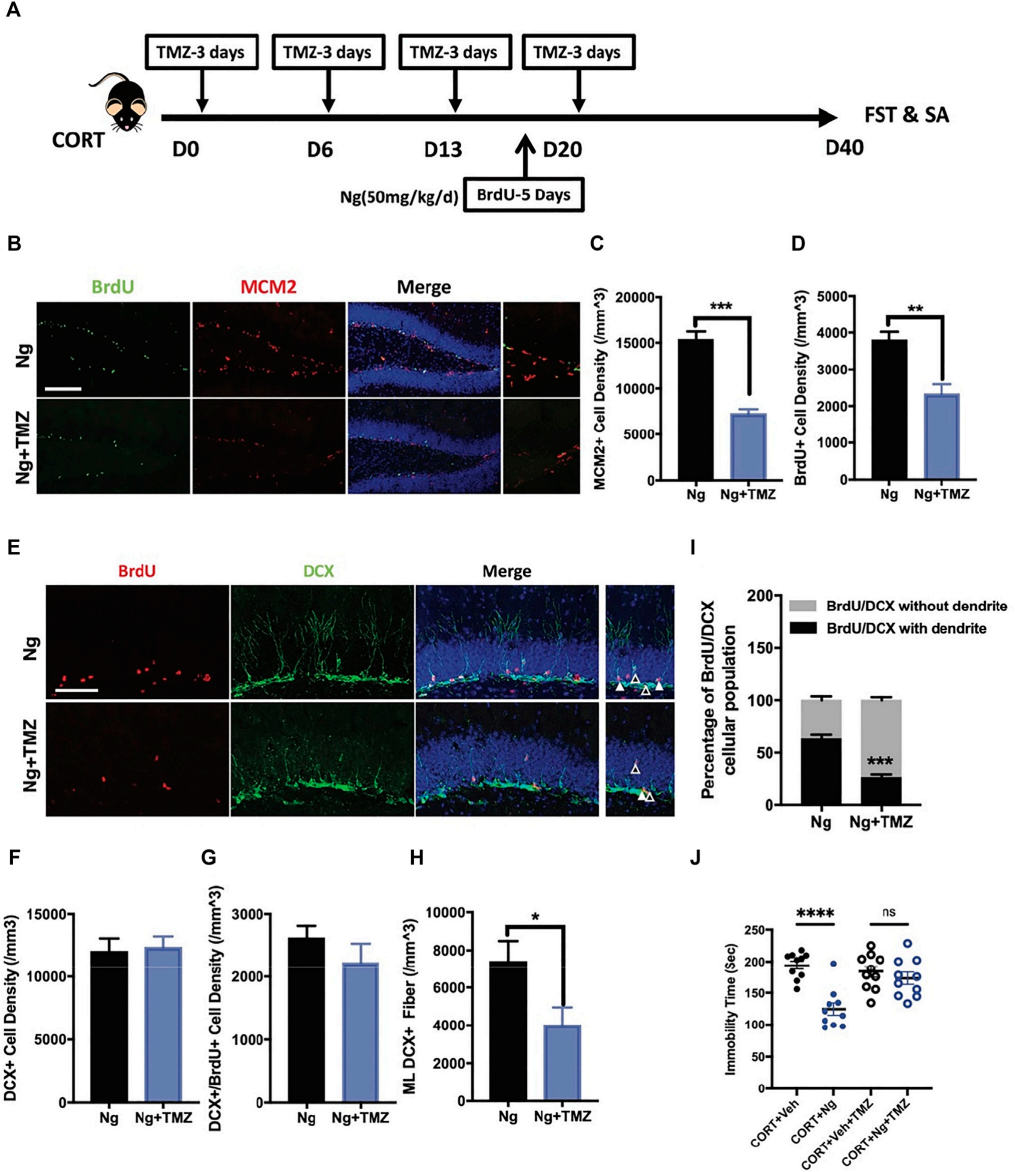
Temozolomide (TMZ), a neurogenic inhibitor, abolishes naringin effects on NSPC differentiation and antidepressant behavior. **(A)** Diagram showing the protocols of BrdU incorporation, Ng treatment and TMZ administration in CORT-induced depression mouse model. **(B)** Representative immunofluorescent imaging results showing the newly proliferative cells (MCM2^+^/BrdU^+^). DAPI was used for nuclear labeling. Scale bar 200 μm **(C)** Statistic analysis of MCM2^+^ cells in the DG region (*n* = 4–5 per group, unpaired student-t test, ***p < 0.001). **(D)** Statistic analysis of BrdU^+^ cells in the DG region. **(E)** Representative fluorescent imaging showing the newborn immature neurons (BrdU^+^/DCX^+^). DAPI was used for nuclear labeling. Scale bar 100 μm. White triangle points for newborn immature neurons with dendritic fiber; empty triangle points for newborn immature neurons without dendritic fiber. **(F)** Statistic analysis of DCX^+^ cells in the DG region. **(G)** Statistic analysis of immature neurons (BrdU^+^/DCX^+^) in the DG region. **(H)** Statistic analysis of DCX^+^ dendritic fiber density in the molecular layer (ML) subregion (*n* = 5 per group, unpaired student-t test, *p < 0.05). **(I)** Statistical analysis of BrdU^+^/DCX^+^ newborn immature neurons (with dendrite/without dendrite). **(J)** Statistical analysis of immobility time in FST. ***N*** = 4–5 per group. *p < 0.05, **p < 0.01, ***p < 0.001.

TMZ is an antimitotic drug as a standard chemotherapy for brain cancer (Gallego 2015; Egeland et al., 2017). TMZ is also an experimental agent used to deplete adult neurogenesis (Fang et al., 2020; Nickell et al., 2020). Herein, TMZ was used to explore the physiological relevance of adult hippocampal neurogenesis to the antidepressant response *in vivo* and *in vitro* (Figure 3A). In the *in vitro* cultured C17.2 cells, both TMZ and CORT significantly inhibited NSC proliferation, showing a decrease in MCM2^+^ and BrdU^+^ cells (Supplementary Figures S1A, S1B). In the *in vivo* experiments, we tested whether the neurogenic effects would contribute to improved immobility for antidepression. Consistent with the *in vitro* study, TMZ treatment blocked the effects of naringin on the MCM2^+^ cells and BrdU^+^ cells in the hippocampus of the CORT-treated mice (Figures 3B–D). Interestingly, TMZ treatment did not affect the density of DCX^+^ immature neurons and the newly generated neuroblasts with DCX^+^/BrdU^+^ staining (Figures 3E–G). Instead, TMZ treatment resulted in a delayed neural commitment of NSPCs. After TMZ treatment, the DCX^+^ dendritic fibers were dramatically decreased in the ML subregion of the DG area (Figure 3H). TMZ treatment decreased the late phase newborn immature neurons with dendritic DCX^+^/BrdU^+^ staining and increased the early phase of newborn immature neurons characteristically with non-dendritic morphology of DCX^+^/BrdU^+^ staining cells (Figure 3I). These data indicate that TMZ could impair the effects of naringin on neuronal differentiation in the adult hippocampal DG region. We further investigated whether TMZ would affect the immobility ability. As expected, FST showed that TMZ treatment abolished the effects of naringin on the immobility time of the CORT-treated mice (Figure 3J). Therefore, the antidepressant effects of naringin could be attributed to the promotion of neuronal differentiation.

### CREB is a Crucial Therapeutic Target for Naringin to Mediate Adult Hippocampal Neurogenesis and Antidepression in CORT-Treated Depression-Like Mice.

**FIGURE 4:**
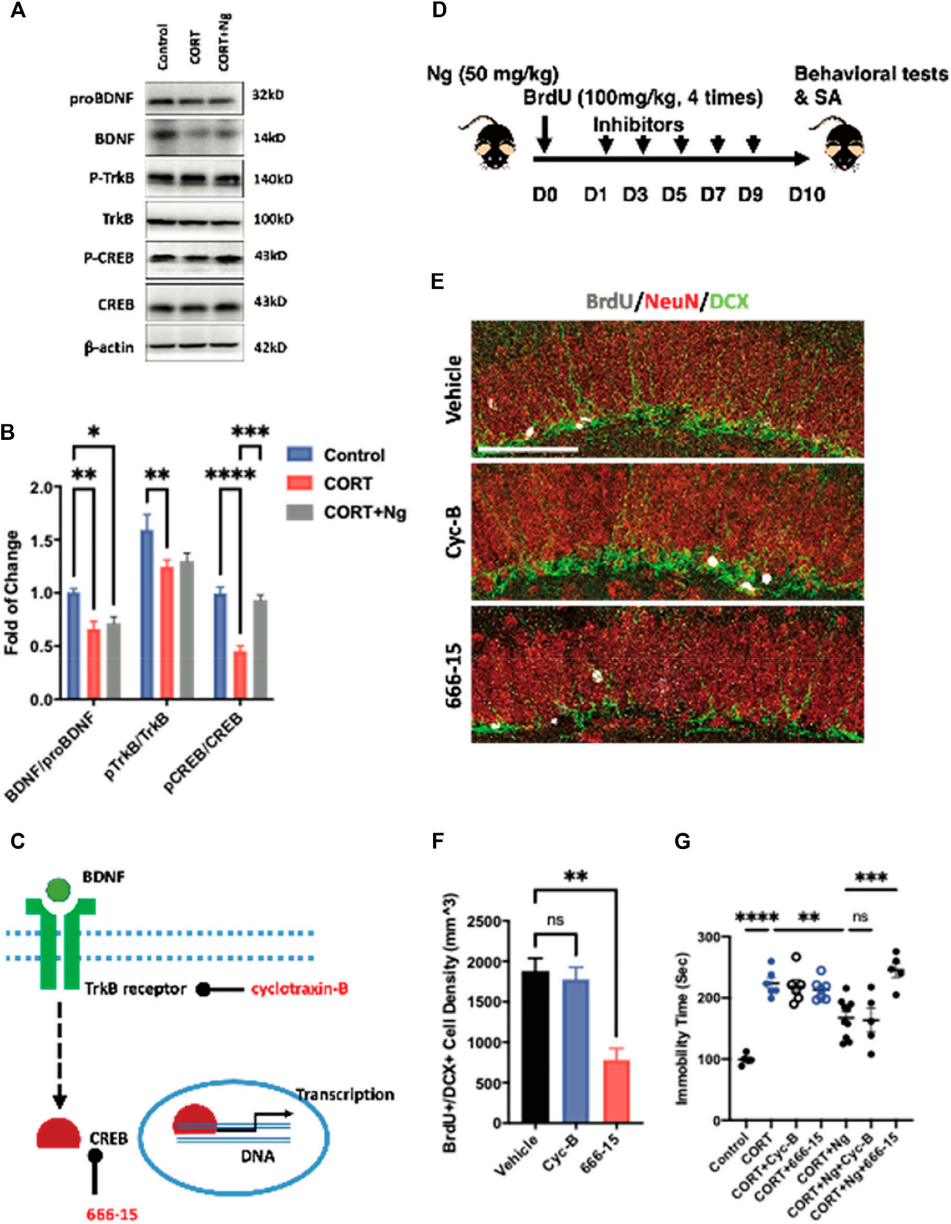
CREB mediates the neurogenic and antidepressant effects of naringin on the CORT-induced depression mice. **(A)** Western blot bands showing the expression of mature and proBDNF, p-TrkB, and TrkB as well as the p-CREB (Ser133) and CREB protein in hippocampus of the CORT-induced depression mice. β-actin was settled as loading control. **(B)** Statistical analysis of mature BDNF/proBDNF, p-TrkB/TrkB level and pCREB/CREB level in the CORT-induced depression mice with or without Ng treatment. **(C)** Diagram showing the specific target of different inhibitors. **(D)** Diagram showing the experimental procedure for treating inhibitor Cyc-B and 666-15, respectively, in Ng-treated mice. **(E)** Representative fluorescent imaging showing newborn immature neurons (BrdU^+^/DCX^+^) in the hippocampal DG region. NeuN was used for labeling mature neurons. Scale bar = 100 μm. **(F)** Statistical analysis on the of BrdU^+^/DCX^+^ cells between vehicle and inhibitor treatment. **(G)** Statistical analysis of immobility time in FST between vehicle and inhibitor treatment. *n* = 5–6 per group. *p < 0.05, **p < 0.01, ***p < 0.001.

BDNF signaling serves as one of the dominant pathways in mediating neural differentiation of NSCs (Sairanen et al., 2005; Li et al., 2008). The BDNF/TrkB/CREB signaling pathway is an important therapeutic target to promote adult hippocampal neurogenesis and attenuate chronic depression (Luo et al., 2017; Peng et al., 2018; Sakata et al., 2013). To understand the underlying mechanisms of antidepressant effects, we examined the expression levels of proBDNF, BDNF, p-TrkB, TrkB, CREB, and p-CREB in the hippocampus region of the normal control (control), CORT plus vehicle group (CORT), and CORT plus naringin (CORT + Ng). The expression levels of BDNF, p-TrkB, and p-CREB were downregulated in the CORT plus vehicle treatment group. Interestingly, naringin treatment had no effect on the expression levels of BDNF and p-TrkB but upregulated the expression of p-CREB in the hippocampus (Figures 4A,B). Those results indicate that CREB could be a target for naringin in promoting adult hippocampal neurogenesis and attenuating depressive behavior.

To explore the roles of the CREB and BDNF receptor TrkB in the naringin-induced neurogenesis, we used 666-15 and cyclotraxin-B (Cyc-B), the specific inhibitors for CREB and BDNF/TrkB, respectively, in the *in vitro* and *in vivo* experiments according to the previous reports (Cazorla et al., 2010; Xie et al., 2017; Xie et al., 2019). In the *in vitro* experiments, both 666-15 and Cyc-B treatment reduced the number of BrdU^+^/MCM2^+^ cells, indicating the inhibition of the NSCs proliferation (Supplementary Figures S1C, S1D). In the *in vivo* study, we investigated the effects of 666–15 (10 mg/kg, i.p.) and Cyc-B (2.5 mg/kg, i.v.) on hippocampal neurogenesis in the CORT-treated mice (Figures 4C,D). Naringin treatment alone was labeled as vehicle in the figures. Consistently, Cyc-B treatment did not change the density of the BrdU^+^ and DCX^+^ cells in the hippocampus of the naringin-treated CORT mice (Figures 4E,F). While the CREB inhibitor 666-15 treatment remarkably compromised the BrdU^+^ and DCX^+^ cells in the hippocampus (Figures 4E,F). Those results strongly suggest that the naringin-induced hippocampal neurogenesis is CREB dependent.

We finally performed the FST to examine the effects of 666–15 and Cyc-B on immobility time of the CORT mice with or without naringin treatment. In the CORT-treated mice, neither Cyc-B nor 666-15 treatment altered the immobility (Figure 4G). In the naringin-treated CORT mice, the 666-15 treatment significantly decreased the immobility time of the mice whereas Cyc-B treatment had no effect on the immobility in the FST (Figure 4G). Taken together, those results suggest that antidepression of naringin could be associated with the promotion of adult hippocampal neurogenesis via activating CREB instead of affecting BDNF production or its receptor response.

## Discussion

Adult hippocampal neurogenesis is essential to build the circuit network with emotional controlling regions such as amygdala and hypothalamus (Fanselow and Dong 2010; Cadiz-Moretti et al., 2016). Continuous generation of functional neurons in adult hippocampus provides structural plasticity to benefit the adaptability in response to environmental stimuli, including psychiatric stressors. In the present study, we examined the effects of naringin on promoting adult hippocampal neurogenesis. The major discoveries include the following: 1) Naringin promoted neuronal differentiation and induced migration of NSPCs into the SVZ-OB system in the normal mice; 2) naringin accelerated neuronal maturation and promoted the dendritic enrichment in the CORT-induced depressive mice; 3) naringin has antidepressant and anxiolytic effects via promoting neuronal differentiation in CORT-induced depressant mice; 4) CREB is a target for naringin to promote neurogenesis and antidepression. To our knowledge, this is the first report where naringin could promote adult hippocampal neurogenesis and exert antidepressant effects via specifically targeting CREB signaling.

Chronic CORT exposure induces depression-like behavior, anxiety, and stress responses via compromising hypothalamic–pituitary–adrenal axis function. As a result, the CORT-induced depression mouse model has been widely used for antidepression studies (David et al., 2009; Myers et al., 2014; Brachman et al., 2016; Gao et al., 2018b; Kinlein et al., 2019; Shu et al., 2019). By immunostaining of the biomarkers for MCM2, BrdU, DCX, and NeuN, we found that naringin could accelerate the process of newborn cells escaping from cell cycle and committing into differentiation stage. Naringin treatment (50 mg/kg/d) did not change the BrdU^+^/DCX^+^ cells but increased BrdU^+^/NeuN^+^ cells in the hippocampus of the CORT-induced depressant mice. After naringin treatment, the increased immature neurons with BrdU^+^/DCX^+^ staining in the hippocampus were only presented at 24 h but not at 7 dpt. Interestingly, the mature newborn neurons charactered by BrdU^+^/NeuN^+^ staining were significantly increased at 7 dpt. Similar effects were also observed in the hippocampal ventral region and olfactory system including SVZ and OB. Naringin treatment remarkably increased the density of DCX^+^ fibers and the DCX^+^ dendritic fibers expanding to molecular layer. Thus, we remark that naringin could induce neuronal differentiation and the maturation of NSPCs for hippocampal neurogenesis.

We next tested the antidepression effects of naringin by FST and TST and anti-anxiety property by OFT. FST and TST are widely used for the antidepression study by assessing the duration of immobility (Porsolt et al., 1977; Gao et al., 2018b). OFT is a commonly used method for anxiety-like behavior assessment by measuring locomotion activity (Gould et al., 2009; Gao et al., 2018b). Naringin treatment reduced immobility in the FST and TST, and prolonged the movement duration at the central region in OFT. The locomotor activity was not affected by both CORT and naringin treatments. These results indicate that naringin has antidepressant and anxiolytic effects. Next, we examined whether the behavior improvement could be attributed to the enhanced hippocampal neurogenesis by using TMZ, a neurogenesis inhibitor (Garthe et al., 2009; Martinez-Canabal et al., 2013; Fang et al., 2020; Nickell et al., 2020). Co-treatment of TMZ inhibited the effects of naringin on NSCs proliferation, neural differentiation, and the dendritic fiber expansion. Coincidently, TMZ abolished the effects of naringin on the immobility time in the CORT depressive mice. Thus, the improved hippocampal neurogenesis could contribute to the antidepressant effects. Notably, a recent study indicates that TMZ could inhibit mtDNA replication and transcription of mitochondrial genes, and induce neural damage in primary rat neural stem/progenitor cells and hippocampal neurons (Lomeli et al., 2020). TMZ could also stimulate mitochondrial biogenesis for myoblast differentiation, induce autophagy, and promote cell fusion (Gatta et al., 2017; Derman et al., 2020). Thus, except for the pharmacological study, the genetic approach is necessary for further study.

The BDNF/TrkB/CREB signaling pathway plays crucial roles in inducing adult hippocampal neurogenesis and attenuating chronic depression (Luo et al., 2017; Peng et al., 2018) (Sakata et al., 2013). BDNF is a therapeutic target to promote survival of neuronal progenitors (Sakata et al., 2013) and induce hippocampal neurogenesis for antidepression (Yau et al., 2011; Jiang et al., 2012). CREB is implicated in the pathogenesis and therapy of depression (Gass and Riva 2007). CREB signaling modulates multiple cellular signaling pathways for antidepression (Odaira et al., 2019). Importantly, an elevated plasma CORT level is correlated with the reduced BDNF level and the impaired hippocampal neurogenesis (Blugeot et al., 2011; Agasse et al., 2020). Thus, we investigated the BDNF/TrkB/CREB signaling pathway. Naringin treatment specifically increased the phosphorylation of CREB but had no effect on the expression of BDNF and the phosphorylation of TrkB. Co-treatment of CREB inhibitor 666-15, rather than BDNF receptor TrkB inhibitor Cyc-B, remarkably reduced the naringin-induced BrdU^+^/DCX^+^ cells in the hippocampus and decreased the immobility time of the CORT-induced depressive mice. Thus, we conclude that CREB signaling might be a critical target for naringin in inducing neurogenesis and antidepression. Of note, naringin was previously reported to upregulate the expression of BDNF and VEGF in rat spinal cord injury model (Rong et al., 2012). The discrepancy of the BDNF responses to naringin treatment could be attributed to different experimental models and dosages.

Overall, those results indicate that naringin is an ideal drug candidate for antidepression treatment. A previous study suggests the safety profile of naringin even used at the dosage up to 500 mg/kg body weight per day and continuously used for 3 and 6 consecutive months (Li et al., 2020). With the long-term safety under high dosage, naringin could be a promising antidepressant agent with clinical application potentials. Nevertheless, we should note the limitations of this study. Firstly, the CORT-induced depressive animal model is not a classic depression model though it is a useful experimental model to connect depression-like behaviors with the deficits of neurogenesis. We need to further confirm the antidepression effects by using chronic unpredictable mild stress model. We also note that the batch-to-batch variations between the behavioral outcome of naringin treatment (Figure 2) and the biological mechanistic study of using different inhibitors (Figure 4). In addition, with the long-term administration of naringin, we should consider the potential artifacts from the stress response to oral gavage. Finally, for data interpretation, we should remark that naringin possesses broad biological activities such as antioxidant (Sharma et al., 2015), anti-inflammation (Luo et al., 2012; Nie et al., 2012), metabolic modulations (Rajadurai et al., 2006), and cholinergic transmission activation (Ben-Azu et al., 2019). The multiple targets would not only contribute to the neuroprotective effects but potentially participate in the neurogenic effects for antidepression.

In conclusion, naringin could promote adult hippocampal neurogenesis and attenuate depression and anxiety via activating CREB signaling in the chronic depression animal model.

## Data Availability

The raw data supporting the conclusions of this article will be made available by the authors, without undue reservation.
