# Supplementary figures and images for: Naringin Mediates Adult Hippocampal Neurogenesis for Antidepression via Activating CREB Signaling
Source: Front Cell Dev Biol. 2022 Apr 7;10:731831. doi: 10.3389/fcell.2022.731831 (PMC9037031; doi:10.3389/fcell.2022.731831)

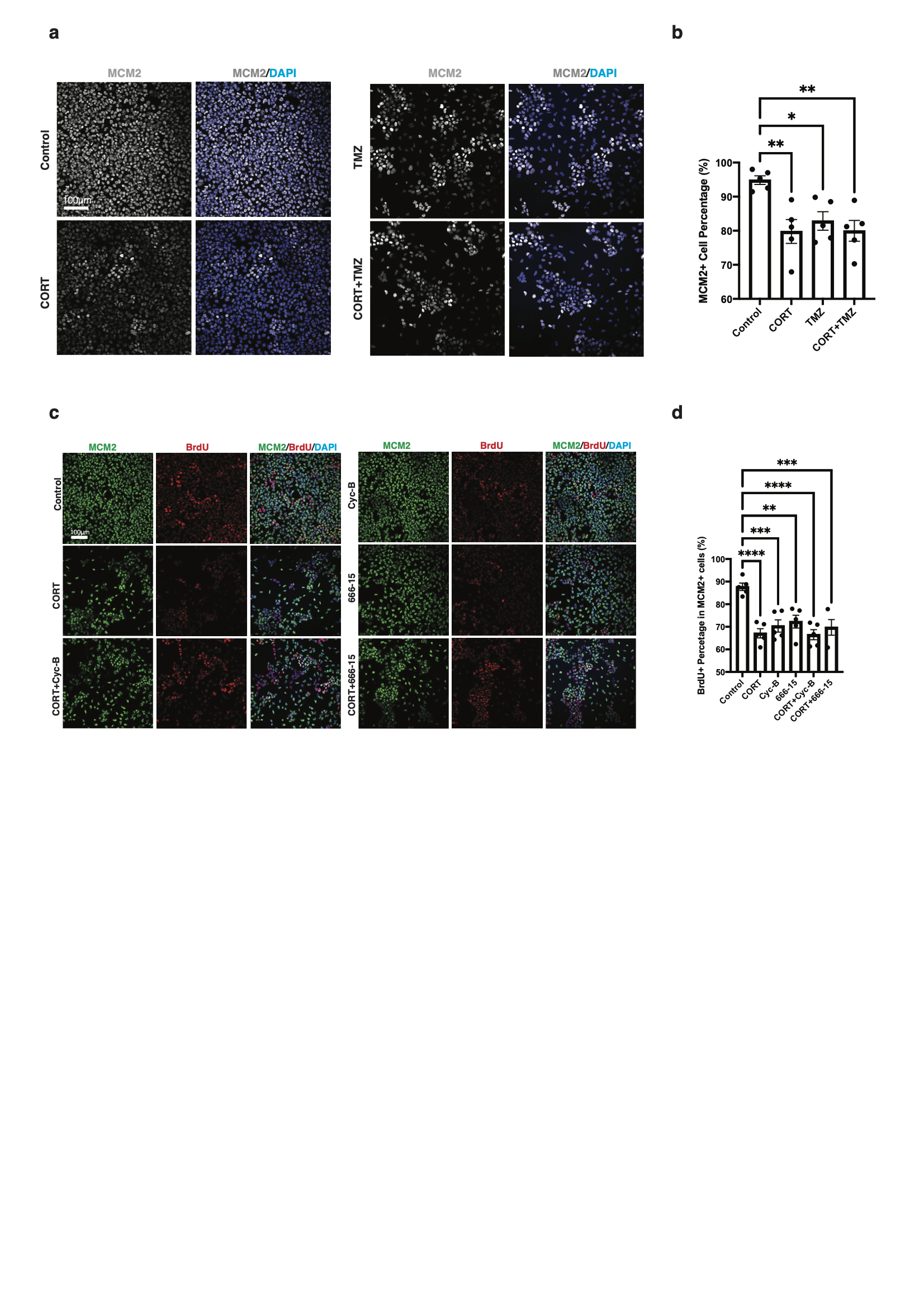

Supplement: Supplementary file 1 [file Image1.TIFF]
