# Supplementary material for: Naringin Mediates Adult Hippocampal Neurogenesis for Antidepression via Activating CREB Signaling
Source: Front Cell Dev Biol. 2022 Apr 7;10:731831. doi: 10.3389/fcell.2022.731831 (PMC9037031; doi:10.3389/fcell.2022.731831)
Supplement: Supplementary file 2 [file DataSheet1.docx]

**Methods of *in vitro* NSCs culture**

C17.2 mouse neural stem cell lines (ATCC) was maintained with DMEM basal medium (Thermo Fisher, US) supplemented with 10% FBS. Cells was seed in the 24-well plate added with round 12mm diameter cover slips. BrdU (10μM, 10mM stock dissolved in DMSO) was added to the cells for 6 hours. Then the chemicals including CORT (10μM), TMZ (5μM), Cyc-B (5μM) and 666-15 ()5μM were incubated with the cells for 24 hours. Cells were fixed with 4% PFA and IF was performed by labelling primary antibodies (Rat-BrdU, Rabbit-MCM2) as well as the secondary antibodies with nuclear labelling with DPAI. Confocal microscope (Zeiss, LSM800) was used for capture image. BrdU^+^ cells percentage in all DAPI labelled cells as well as the BrdU^+^ cells in all MCM2^+^ cells were recorded for reflecting the cell growth and cell proliferation speed, respectively (Supplementary Figure). Fiji software was used for cell counting analysis.

**Supplementary figure legends**

**Sfig1: The effects of TMZ, Cyc-B and 666-15 to cultural NSCs. a:** Confocal image to show the positive pattern of the BrdU (Gray) and nuclear labelling (DAPI, Blue). **b:** Statistical analysis of the BrdU^+^ percentage in all DPAI^+^ cells (unpaired students’ t-test, p<0.01). **c:** Confocal image to show the positive pattern of MCM2 (Green), BrdU (Red) co-labelled with DAPI (Bule) in cultural NSCs. d: Statistical analysis to show the different BrdU^+^ percentarge in all MCM2^+^ cells (one-way ANOVA, Tukey’s post-hoc test, **p<0.01, ***p<0.001).
